# Group-Level EEG-Processing Pipeline for Flexible Single Trial-Based Analyses Including Linear Mixed Models

**DOI:** 10.3389/fnins.2018.00048

**Published:** 2018-02-06

**Authors:** Romy Frömer, Martin Maier, Rasha Abdel Rahman

**Affiliations:** ^1^Cognitive Linguistic and Psychological Science, Brown University, Providence, RI, United States; ^2^Humboldt-Universität zu Berlin, Berlin, Germany; ^3^Berlin School of Mind and Brain, Humboldt-Universität zu Berlin, Berlin, Germany

**Keywords:** EEG, EEGLab, Linear mixed models, cluster-based permutation tests, processing pipeline

## Abstract

Here we present an application of an EEG processing pipeline customizing EEGLAB and FieldTrip functions, specifically optimized to flexibly analyze EEG data based on single trial information. The key component of our approach is to create a comprehensive 3-D EEG data structure including all trials and all participants maintaining the original order of recording. This allows straightforward access to subsets of the data based on any information available in a behavioral data structure matched with the EEG data (experimental conditions, but also performance indicators, such accuracy or RTs of single trials). In the present study we exploit this structure to compute linear mixed models (LMMs, using lmer in R) including random intercepts and slopes for items. This information can easily be read out from the matched behavioral data, whereas it might not be accessible in traditional ERP approaches without substantial effort. We further provide easily adaptable scripts for performing cluster-based permutation tests (as implemented in FieldTrip), as a more robust alternative to traditional omnibus ANOVAs. Our approach is particularly advantageous for data with parametric within-subject covariates (e.g., performance) and/or multiple complex stimuli (such as words, faces or objects) that vary in features affecting cognitive processes and ERPs (such as word frequency, salience or familiarity), which are sometimes hard to control experimentally or might themselves constitute variables of interest. The present dataset was recorded from 40 participants who performed a visual search task on previously unfamiliar objects, presented either visually intact or blurred. MATLAB as well as R scripts are provided that can be adapted to different datasets.

## Introduction

If I do something 100 times, will every time be the same? If I classify fruits as apples and pears, how does the appearance of a particular fruit influence how easily I can identify it as one or the other? How does my experience in classifying apples and pears shape my brain responses to this task? How does variability in neural responses relate to variability in behavior?

Addressing such questions is facilitated by recent developments in statistical and signal processing methods, paralleled by the emergence of open source toolboxes implementing those methods. In many disciplines, ANOVAs, comparing means between conditions at the level of participant averages are now complemented by regression-based methods, such as linear mixed models (LMMs, e.g., Baayen et al., 2008) estimating manipulation-related trial-by-trial variations in behavior and neural correlates. Open source toolboxes, such as EEGLAB (Delorme and Makeig, 2004), allowing for flexible, easy and transparent access to the data, facilitate the application of such methods to psychophysiological data (Dambacher et al., 2006; Dimigen et al., 2011).

## Why change a winning team? limitations of traditional averaging approaches and solutions

Typically, after preprocessing (re-referencing, ocular correction, filtering, segmentation, baseline correction, and artifact rejection) EEG data are averaged within conditions and participants, and these averages are then analyzed for mean differences between conditions and their interactions using repeated measures ANOVAs. Averaging serves to extract the event-related potential (ERP) from background activity. This traditional averaging approach has several limitations. One of these limitations is related to the implicit assumption that every participant's average has the same quality and that the same number of observations constitutes each of those averages. In practice EEG datasets often do not meet this assumption even when equal numbers per cell are experimentally planned. Differences in performance accuracy and artifact rejection during EEG-data processing inevitably result in unequal numbers of trials contributing to individual averages within participants and conditions. These unequal contributions are not considered in traditional ANOVA approaches, where every participant's average has the same weight in all conditions. A second limitation is related to the implicit assumption that experimental manipulations yield uniform effects across all participants and items. Random variance (individual differences or variance across items) in effect sizes are not taken into account. In reality, participants and items may vary substantially in their effect sizes, which can lead to biases in group-level estimates. The most severe limitation of the averaging approach is its dependency on discrete factor levels and its resulting inability to test for parametric effects. Splitting continuous variables into categorical variables reduces statistical power and might conceal nonlinear effects with results critically depending on the range in which variables were sampled and the way the variables were split (Cohen, 1983; MacCallum et al., 2002; Baayen, 2004). Furthermore, splitting and the required matching of other variables may result in a selection of unusual materials (Hauk et al., 2006).

As demonstrated by Smith and Kutas (2015a), as an alternative to averaging, ERPs can be estimated using regression procedures and in fact the averaging method is merely a special case of the least squares method underlying regression. Table 1 provides an overview of the commonalities and differences of ANOVA, regression and linear mixed models (LMMs). LMMs, like regression and ANOVA, are based on the general linear model (for an overview, see Bolker, 2008). In addition to estimating regression weights (or contrasts) at the group level—*fixed effects*—they also estimate systematic variance between individuals—*random effects*. To put it simply, they jointly estimate group effects and individual differences—the latter often considered nuisance in experimental approaches (Cronbach, 1957). Fixed effects include the intercept (*b*_0_ in regression terms), often the grand mean across all participants and conditions (depending on contrast settings), and effect estimates for each predictor (i.e., experimental condition or covariate) and specified interaction terms. Random effects provide estimates for the variance of these effects. Not formally parameters of the model, Best Linear Unbiased Predictors (BLUPS) are also provided that estimate how each participant (or item) systematically varies from those group level estimates (Baayen et al., 2008). Thus, for each specified random effect, BLUPS are individual participants' estimates of those effects relative to the group estimate and can be read out from the model (examples for BLUPS will be provided in brackets along with the explanation of the corresponding different random effects). Random effects include as a minimum *random intercepts*, that is how much individuals differ from the group intercept (the group level P3 component might have an amplitude of 4.2 μV, but R.F.'s mean amplitude might be 6.3 μV [+2.1 μV], while M.M.'s is 2.7 μV [−1.5 μV] and R.A.R's is 3.6 μV [−0.6 μV]). These can also be specified for items, allowing the dependent variable to vary between different stimuli (e.g., the P3 component to different apples and pears might vary depending on how prototypical a given item is for the respective category). When random effects are estimated for both individuals and items, that is referred to as crossed random effects (Baayen et al., 2008). Finally, random effects can be specified for experimental effects—as *random slopes*, both by-subject or by-item. These estimate to which degree experimental effects vary between individuals or items (e.g., R.F. might show a stronger effect of an uncertainty manipulation compared to the group mean while M.M. might show a smaller effect). Accounting for these additional sources of variance provides more reliable group-level estimates (Barr et al., 2013; Matuschek et al., 2017), but these variations might as well be exploited to investigate how individual differences in experimental effects relate to each other (random effect correlations; Kliegl et al., 2010). With these features, LMMs are a powerful tool to overcome the limitations of traditional averaging approaches in ERP research (see above):

**Table 1:** Comparison of methods.

	**ANOVA**	**Regression**	**LMM**
Dependent variable format	By condition and participant averages	Single trials	Single trials
Predictors	Only categorical	Categorical and continuous	Categorical and continuous
Random intercept per participant	Yes	Not standard	Yes
Crossed random effects	No	No	Yes
Random slopes	No	No	Yes

First, LMMs—performed on single trials—take all data into account and are robust to unequal numbers of observations per cell and even missing values (Pinheiro and Bates, 2000). They are therefore suitable for unbalanced designs, e.g., where participants' behavior determines the number of observations (Fröber et al., 2017). Second, random slopes provide a means to estimate *random* variance in effect sizes while computing the *fixed* group effect, yielding more robust estimates and avoiding Type I errors (Barr et al., 2013; Matuschek et al., 2017). Third, in contrast to the many observations of limited discrete sampling points of the independent variable required by the averaging approach, regression based approaches with fewer observations of discrete values across a larger value range have larger power and allow for the test of nonlinear relationships (Cohen, 1983; MacCallum et al., 2002; Baayen, 2004). To illustrate, when investigating the effect of reward on feedback related potentials, in order to obtain reliable ERPs for each type of reward one would need at least 30 trials of each reward magnitude. On the other hand, in a regression-based approach, on the same overall number of trials, reward could be varied continuously from a given low to a given high reward, and linear, but also quadratic or cubic effects of reward across that range could be tested (Frömer et al., 2016a).

To summarize, single-trial regression-based approaches are equivalent to the averaging approach with regard to estimating ERPs (Smith and Kutas, 2015a). Further, LMMs as an extension of standard regressions help account for some of the problems posed for the application of ANOVAs, such as unequal observations per cell (Pinheiro and Bates, 2000), variability in effect sizes across individuals (Barr et al., 2013; Matuschek et al., 2017) and items (Baayen et al., 2008), as well as unbalanced designs (Fröber et al., 2017). We will next explore some examples where the advantages of LMMs (as an instance of the regression based approach) were successfully exploited to investigate ERPs.

LMMs were first applied to EEG data in 2011 (Amsel, 2011; Dimigen et al., 2011), their application to psychophysiological data was proposed long before that (Bagiella et al., 2000). In the psycholinguistic domain, LMMs are widely used, following the maxim that “words are people, too” (R. Kliegl, personal communication) in the sense that, just like people, they vary in a myriad of characteristics. Amsel (2011) investigated the effects of a variety of such characteristics, e.g., semantic richness and imageability on ERPs related to word processing, while controlling for variables well known to affect word processing, such as word length and word frequency and crossed random effects. Dimigen et al. (2011) used LMMs to control for word and sentence characteristics and crossed random effects in sentence reading with simultaneous EEG and eye tracking, showing effects of gaze duration, word predictability and frequency on N400 amplitude. Since, LMMs have been applied in active reading, to ERPs (Kornrumpf et al., 2016) and in the time-frequency domain (Kornrumpf et al., 2017), in the motor learning domain (Frömer et al., 2016a,b) and in the area of cognitive control (Fröber et al., 2017).

These are just a few selected examples meant to illustrate the potentials of this approach. They are certainly not the only studies applying LMMs, and other regression based approaches have been applied and for example been combined with computational modeling (e.g., Cavanagh et al., 2012; Fischer and Ullsperger, 2013; Collins and Frank, 2016).

## The “what” to the “how”: determining time windows and regions of interest

Choosing a statistical approach is only one decision researchers have to make. Another, and perhaps more difficult decision is what data to apply this approach to. EEG data are incredibly rich. In the present dataset, we have 65 channels and 1s of time series at each of those, summing up to 26,000 data points to be potentially analyzed (the 200 ms baseline excluded). This number is not unusual for EEG experiments. Thus, determining the right time-windows and regions of interest (ROIs) to test group-level effects is a challenge. Even with clear hypotheses on which ERP components will be affected by the experimental manipulations, the selection is not trivial, because latencies and topographical distributions can vary to a certain degree across studies. The problem is amplified when new effects are explored and there is no substantial previous literature to help develop direct hypotheses on the nature of those effects (e.g., time course or spatial distribution). Especially in face of the replication crisis, fishing expeditions that might yield some, but in the worst-case spurious effects, should be avoided (for a detailed discussion, see Luck and Gaspelin, 2017). One statistically robust way to determine suitable time windows and electrode sites are cluster-based permutation tests (CBPT) as implemented in FieldTrip (Maris and Oostenveld, 2007). In a nutshell, this approach tests the null hypothesis that observations for different conditions are drawn from the same distribution and are therefore exchangeable. Therefore, if observing similar effects under random assignment of condition labels is highly unlikely (less than 5% of the permutations show them), this hypothesis is rejected and the observed condition effect is considered significant (Maris, 2004, 2012). The cluster-based procedure further makes use of the EEG property that observations on adjacent sites and time points are often correlated, because a real effect most likely affects multiple electrodes similarly and persists across several tens to hundreds of milliseconds (or sampling points). While in other approaches this violates assumptions of statistical independence, here this property is exploited to identify spatio-temporal clusters. Samples with positive and negative *t*-values (retrieved from simple *t*-tests at each sensor-sample pair) exceeding a threshold (e.g., *p* < 0.05 according to parametric test) are clustered separately. The added *t*-values of sampling points within each cluster form the cluster-level statistics. The largest (absolute) value from that cluster level statistic is then compared to the permutation distribution of maximal cluster statistics. That permutation distribution is created by randomly assigning condition labels and running the same test many times (e.g., 1,000 times), retrieving the maximum cluster statistics every time. If the maximum cluster statistic from the real data is larger than 95% of the maximum cluster statistics in the permutation distribution, then the null hypothesis that the two conditions are sampled from the same distribution is rejected. To temporally and spatially locate the effect, all (absolute) cluster level statistics larger then the 95th percentile (for α = 0.05) of the permutation distribution are then taken to be significant under the assumption that no cluster statistic exceeds this critical value. The major advantage of this approach is that it does not require knowledge or assumptions about the underlying unknown distributions and that it reduces a large quantity of comparisons down to one statistical test, reducing Type I error probability while maintaining sensitivity. A detailed description of the procedure and mathematical demonstration of its correctness are provided in Maris and Oostenveld (2007).

## The present study

**Figure 1:**
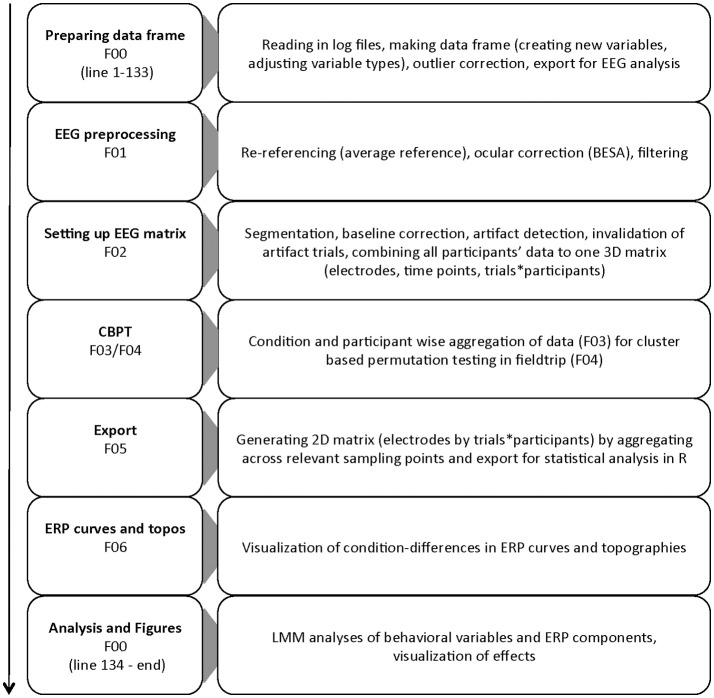
Flow chart of the different parts of the processing pipeline. Script names are reduced to their numbers for simplicity.

The goal of the present study is to facilitate the application of regression-based methods on single-trial ERP data by providing example code covering the whole process from data cleaning to statistical analysis with LMMs. Thus, we present an application of a processing pipeline integrating (1) EEG-data processing with EEGlab (Delorme and Makeig, 2004) for data cleaning, structuring and plotting, (2) applying cluster-based permutation-tests as implemented in FieldTrip (Maris and Oostenveld, 2007) for data screening and ROI selection, and (3) single-trial based LMM analyses using the lme4 package for R (Bates et al., 2015b). An overview of the processing pipeline is shown in Figure 1.

The data were obtained using a visual search task, in which participants indicated the location of a deviant object in a circular array of newly learned objects (left or right). Search arrays were either presented intact or blurred, manipulating perceptual certainty. Besides typical analyses of manipulation effects on ERPs, here we link single-trial estimates of behavior and neural correlates of decision-making—particularly N2 and P3b —to demonstrate how the presented pipeline can also be employed to investigate brain-behavior relationships. All data and scripts can be downloaded from https://osf.io/hdxvb/.

## Methods

### Participants

The final sample comprised 40 participants (8 male) with a mean age of 23.82 years (*SD* = 5.07). Participants gave informed consent and received course credits for their participation.

### Apparatus and stimuli

Stimuli were presented on a 4/3 17″ BenQ monitor with a resolution of 1280 x 1024 using Presentation (Neurobehavioral Systems, Berkeley, USA) at a viewing distance of 60 cm. Stimuli consisted of eight rare, unfamiliar objects presented on a light blue square producing an equal stimulus size of 2.7° visual angle for each object stimulus. Each stimulus array contained 12 objects (all either intact or blurred) arranged in a circle (diameter = 12° visual angle). For blurred stimuli, a Gaussian filter (sigma = 10) was applied to the original stimuli. Manual responses and RTs were registered using custom-made response buttons.

### Procedure and design

**Figure 2:**
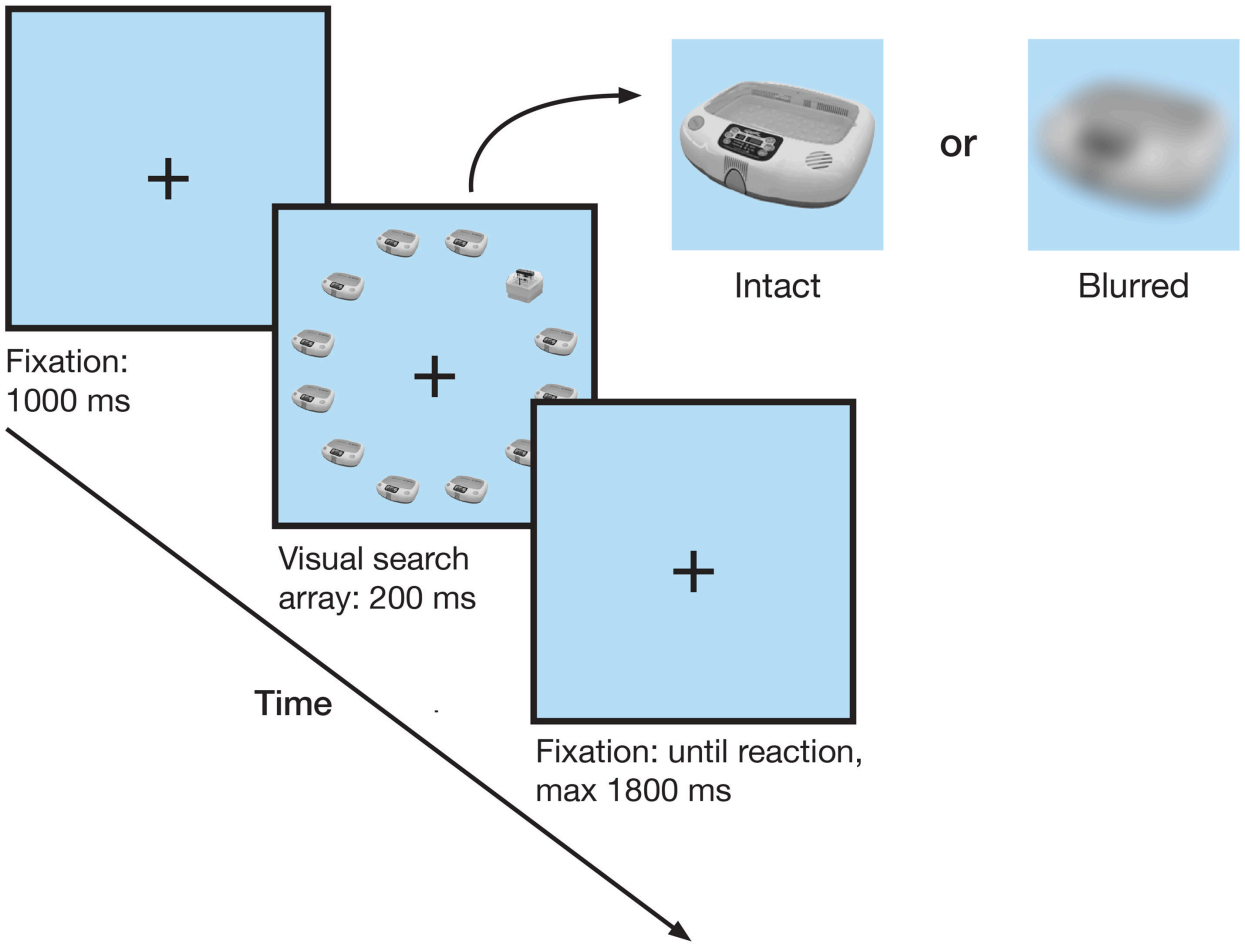
Trial schematic. After a 1 s fixation cross, a circular visual search array of 12 objects was shown. Participants indicated whether the deviant object was on the left or the right side. Trials with the deviant in the top or bottom row were not analyzed.

Participants performed a visual search task while EEG was recorded. After a short practice block participants performed 1920 trials of the visual search task, organized in 5 blocks separated by self-paced breaks. Each trial started with the presentation of a fixation cross at the center of the screen, followed by the search display after 1 s. The search display consisted of 11 identical objects and one deviant, and was presented for 200 ms, followed by a fixation cross. Participants indicated whether the deviant object was on the left or right side of the display by pressing a button with their corresponding hand's index finger (Figure 2). Within a search display all stimuli were visually similar (either light or dark stimulus group), and presented either intact or blurred. Trials ended with the response or after a time out of 2 s after search array onset. Prior to the test session, participants had acquired knowledge about the objects (Maier et al., 2014), which will not be investigated here.

### Electrophysiological recording and analyses

EEG data were recorded using brain vision recorder (Brain Products) from 64 Ag/AgCl electrodes mounted in standard electrode caps (Easycap) with a sampling rate of 500 Hz, and referenced against A1. The vertical EOG was recorded below the left eye (IO1).

EEG data analysis was conducted using Matlab (R2016a, MathWorks Inc.) and the EEGlab toolbox (Version 13_6_5b; Delorme and Makeig, 2004). EEG data were re-referenced to average reference and A1 activity was retrieved. Ocular artifacts were corrected with surrogate data based on individual eye movements recorded separately and obtained using Brain Electric Source Analysis (BESA 6.0) software (Ille et al., 2002). The corrected data were filtered (0.5 Hz low cut off and 40 Hz high cutoff). These steps were performed using F01_preprocessing.m. Cleaned data were segmented from −200 to 800 ms relative to stimulus onset and baselines were corrected to the prestimulus interval. Segments containing artifacts, hence values ±150 μV or gradients larger than 50 μV were invalidated and thereby excluded from further analysis. All trials of all participants were combined in a 3D matrix (channels, time points, trials by participants), which forms the basis for all further ERP analyses (F02_epoching_structuring.m).

### Analyses

Behavioral data processing and statistical analyses other than CBPT were conducted using R-Studio (Version 3.1.1; R Core Team, 2014). Trials in which the deviant was on the center-top or center-bottom positions and therefore hard to assign to either the left or right visual field, and miss trials were excluded from all analyses. Accuracy was analyzed with generalized linear mixed models (GLMMs), fitting a binomial model (Bolker, 2008). For the linear mixed models (LMM) analyses of reaction times (RTs) and ERPs, we further excluded trials with incorrect responses. LMMs and GLMMs were computed with the lme4 package (Bates et al., 2015b) and *p-*values with the lmerTest package, using Satterthwaite approximation for degrees of freedom. RTs were modeled using perceptual certainty (intact vs. blurr) and deviant position (right vs. left visual field) as fixed effects. As random effects we modeled random intercepts for participants (variance in individual means across all conditions, e.g., variance in average response time or ERP magnitude) and object pairs (variance in means across stimuli, e.g., variance in average response times across stimuli), as well as random slopes for the predictors (estimates the variance in the effect of a given manipulation across individuals or items). Random effects not supported by the data, that is explaining zero variance according to singular value decomposition were excluded to prevent overparameterization (Bates et al., 2015a). For all predictors we applied sliding difference contrasts, thus the resulting estimates can be interpreted as the difference between subsequent factor levels (level 2 minus level 1, e.g., intact minus blurred). The advantage of this contrast is that the fixed effect intercept (group-level mean) is estimated as the grand average across all conditions (e.g., the empirical group-level mean), rather than the mean of a baseline condition, as for example for the default treatment contrast, which can cause troubles when using multiple predictors. Single trial information for CBPT and plotting was exported to Matlab in the same analysis script, F00_behavioral_data_and_LMM_analyses.R. CBPT were performed using FieldTrip (Version 20170701) in F04_permutation tests.m based on aggregated data obtained with F03_prep_permutation_tests.m. Relevant time windows of the single trial EEG data, as determined using CBPT were then exported for single trial LMM analyses using F05_export.m (see section electrophysiology in results for specific time windows and regions of interest). ERP data were plotted using F06_plotting.m. Fixed effects structures of LMMs and GLMMs were reduced stepwise by excluding non-significant interaction terms/predictors and compared using anova ratio tests until the respectively smaller model explained the data significantly worse than the larger model (significant *X*^2^-test). We further compared and report AIC (Akaike Information Criterion) and BIC (Bayesian Information Criterion), fit indices that are smaller for better fitting models. Compared to AIC, BIC implements a stronger penalty for model complexity (number of parameters). Significant interactions would be followed up by running models with factors constituting the interaction within each other to obtain estimates for the comparison within each level of the respective other factor. Note that for this procedure to be accurate, these models need to be specified identically to the original model except for the nesting. For comparison, we also provide code to obtain these with the difflsmeans function from the lmerTest package.

## Results

### Behavior

**Figure 3:**
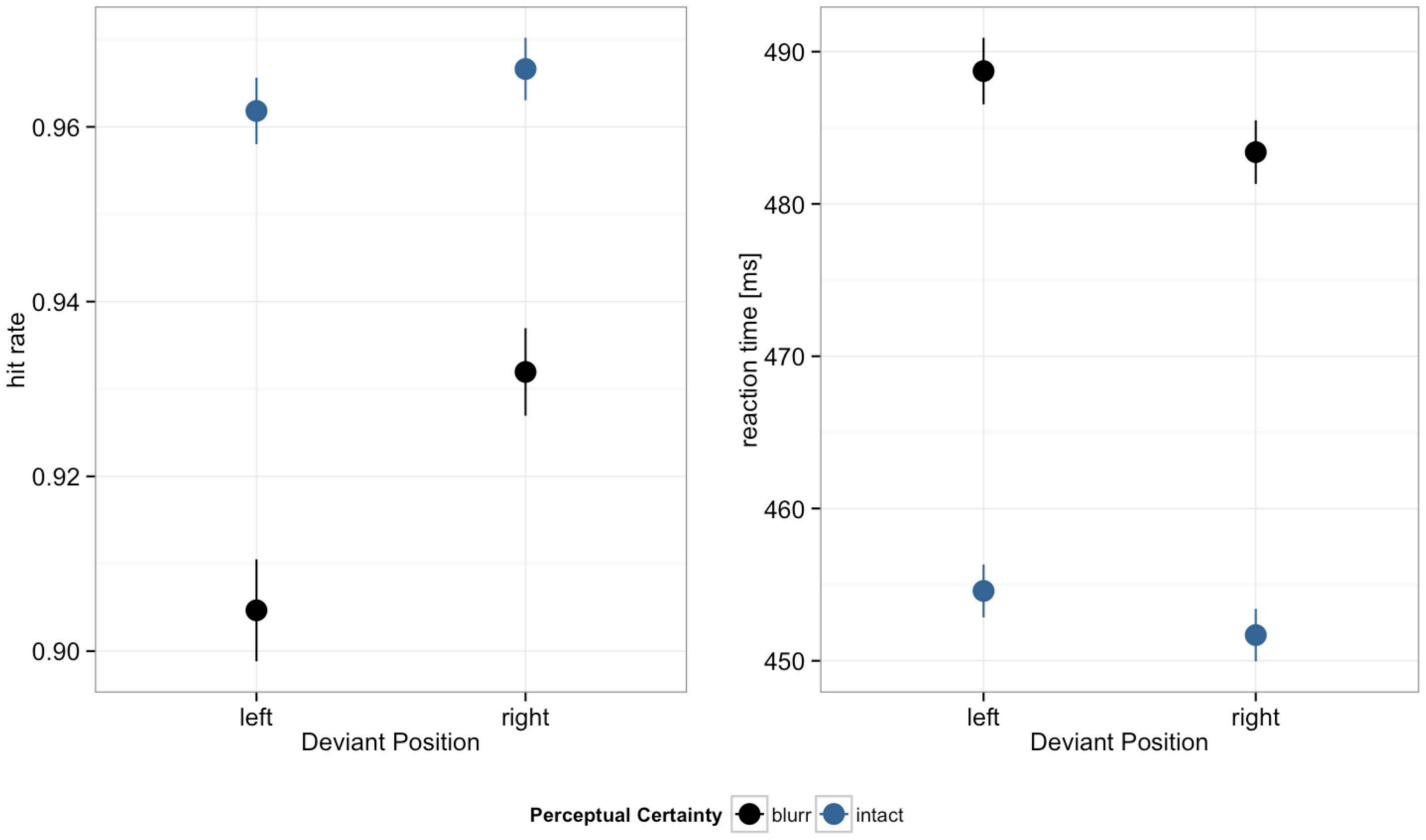
Hit rates and reaction times by deviant position and perceptual certainty. Error bars depict 95% confidence intervals (CI).

Hit rates and reaction times are displayed in Figure 3. Reduced perceptual certainty impaired performance, decreasing hit rates and increasing reaction times. Hit rates were further higher in the right compared to the left visual field. In both hit rates and RTs we observed significant perceptual certainty by deviant position interactions. Model estimates are summarized in Table 2.

**Table 2:** Effects of perceptual certainty on performance contingent on deviant position.

**Variable**	**Accuracy**					**Reaction time**				
	***b***	***SE***	***z*-value**	***p*-value**		***b***	***SE***	***t*-value**	***p*-value**	
Intercept	3.31	0.19	17.45	<0.001	^***^	469.85	9.93	47.34	<0.001	^***^
PC i - b	0.96	0.14	6.92	<0.001	^***^	−33.94	5.98	−5.68	<0.001	^***^
DP r - l	0.32	0.11	2.76	0.006	^**^	−4.72	3.42	−1.38	0.174	
PC: DP	−0.19	0.09	−2.17	0.030	^*^	3.71	1.65	2.26	0.024	^*^
**Variance components**	***SD***	**Goodness of fit**				***SD***	**Goodness of fit**			
Participants	0.89	Log likelihood		-9963		53.01	Log likelihood			−281084
PC	0.35	REML deviance		19926		14.30	REML deviance			562168
DP	0.52				16.61					
Stimulus	0.49				21.20					
PC	0.45				21.89					
DP	0.22				8.10					
Residual					89.57					

*PC, Perceptual Certainty (intact – blurred); DP, Deviant Position (right – left)*.

*** *p < 0.001*,

** *p < 0.01*,

* *p < 0.05*.

The upper part of the table displays the fixed effects for the GLMM (left, accuracy) and LMM (right, RTs). GLMM estimates are log-ratios and LMM estimates can be read out directly in milliseconds. Note that in these analyses we only use categorical predictors with two factor levels each applying sliding difference contrasts, thus the estimates refer to the differences in means (or changes in log-ratios in response types for GLMMs) between factor levels (or conditions). The bottom part of the table summarizes the random effects, providing standard deviations as estimates of the variance in each component (Participant and Item are random intercepts, respectively and indented components below are the corresponding random slopes for perceptual uncertainty and deviant position), and goodness of fit estimates log likelihood and restricted maximum likelihood (REML) deviance. We report these metrics in line with the documentation in Kliegl et al. (2013).

We followed up the significant interaction by running additional models with the two factors nested within each other to obtain the effects of one factor at each level of the other factor, respectively. For accuracy, the model with deviant position nested within perceptual certainty revealed a significant deviant position effect for blurred (*b* = 0.41, *p* < 0.001), but not intact stimuli (*b* = 0.22, *p* = 0.085). Nesting perceptual certainty within deviant position, we obtained significant effects for both, left (*b* = 1.05, *p* < 0.001) and right (*b* = 0.86, *p* < 0.001) deviant position. In RTs, there was no significant deviant position effect for either blurred (*b* = −6.57, *p* = 0.067) or intact stimuli (*b* = −2.86, *p* = 0.418). When nesting perceptual certainty within deviant position, perceptual certainty effects were significant for the left (*b* = −35.80, *p* < 0.001) and right (*b* = −32.08, *p* = 0.001) deviant position.

For comparison, we ran standard repeated measures ANOVA and regression on the RT data (cf. F00 for detailed outputs of those analyses). The ANOVA showed only a main effect of perceptual certainty, while the regression showed significant main effects for both perceptual certainty and deviant position. We compared AICs and BICs of regression and LMM to assess relative fit to the data and the LMM had smaller, hence more favorable fit indices than the regression (AIC: 565152 vs. 578335, BIC: 565249 vs. 578379). To see what drives the difference between LMMs and regression, we ran two additional LMMs: one omitting the random effect for items and one with random intercepts per participant only, omitting random slopes. Both models still showed significant interactions of perceptual certainty and deviant position. The main effect of deviant position emerged as a trend in the model without crossed random effects and became significant when omitting random slopes. The standard error of that effect dropped from 3.42 in the original model to 2.69 in the model without crossed random effects to 0.86 in the model with random intercept for participants only, which is how the small effect yielded significance. Thus, the LMM was more sensitive than the ANOVA and more specific than the ordinary regression.

For accuracy, ANOVA is not the appropriate test, so we only ran a logistic regression in comparison. The logistic regression on accuracy yielded the same results as the LMM with regard to significant effects, however, similar to the RT models, the regression underestimated the standard errors. Further, AIC and BIC for the GLMM were smaller than those for the logistic regression (AIC: 19958 vs. 21938, BIC: 20100 vs. 21974), indicating that the GLMM fits the data better. Again, we stepwise omitted random effects in the GLMM and standard errors of the estimates approached those in the logistic regression (and so did AIC and BIC).

### Electrophysiology

**Figure 4:**
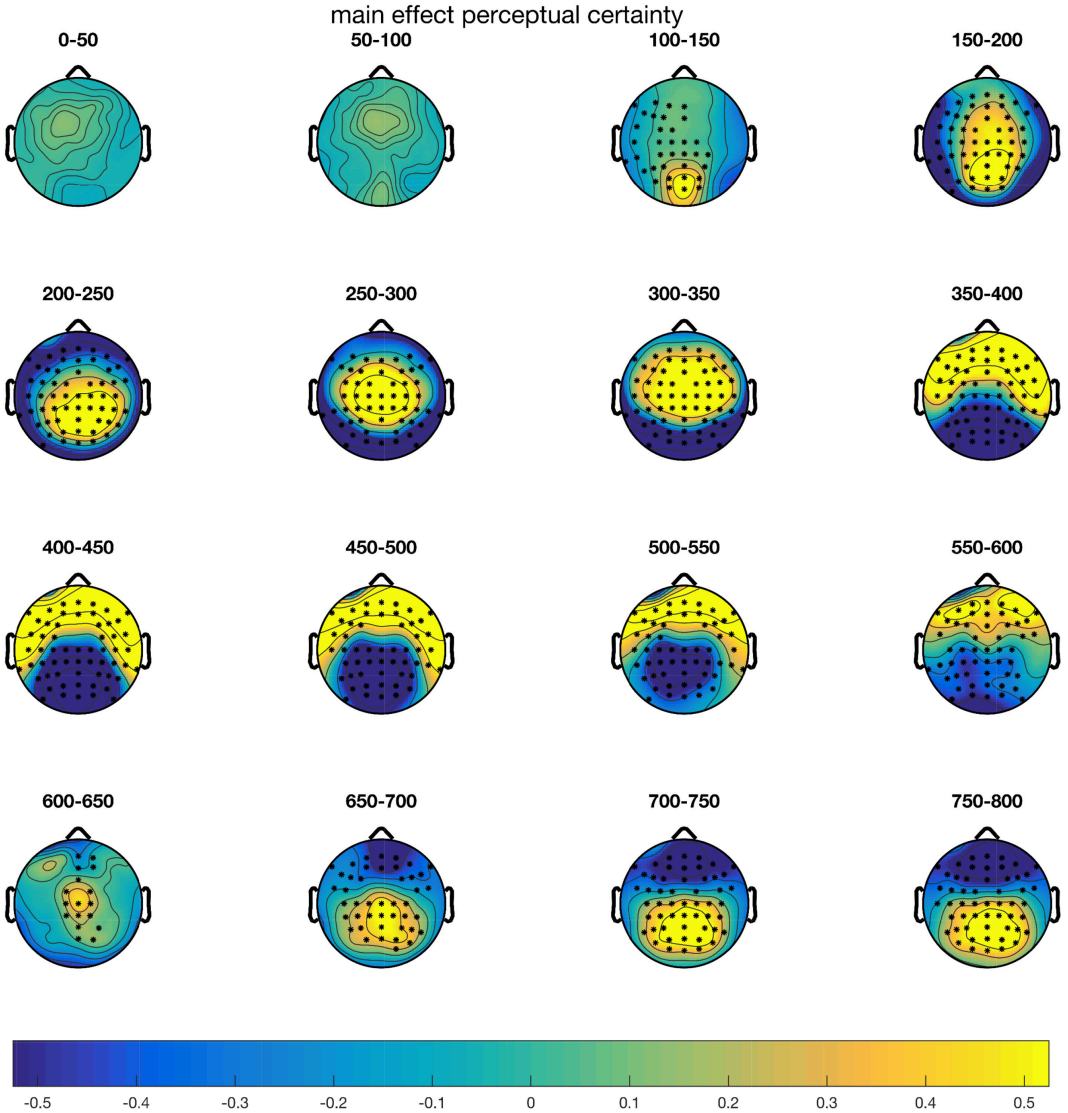
Results of the CBPT on the main effect of perceptual certainty (blurr–intact). Electrodes that are part of clusters with *p*-values < 0.05 are highlighted in the corresponding time windows.

CBPTs comparing mean amplitudes over the epoch 0–800 ms revealed that the blurred and intact stimulus conditions differed significantly. As shown in Figure 4, differences started around 100 ms after stimulus onset and remained throughout the whole epoch. Three clusters underlay the significant difference: From 116 ms on, the blurred condition evoked more positive amplitudes at central, parietal and frontocentral electrode sites (*p* = 0.002). Furthermore, two clusters with lower amplitudes in the blurred compared to the intact condition were observed, between 124 and 594 ms at parietal and occipital electrodes (*p* = 0.002) and between 640 and 800 ms at frontal and frontocentral electrodes (*p* = 0.008). Here we limit the follow-up analyses to the fronto-central N2 between 250 and 350 ms (FC1, FC2, C1, Cz, C2), as well as the centro-parietal P3b/CPP (CP3, CP1, CPz, CP2, CP4, P3, Pz, P4, PO3, POz, PO4) between 400 and 550 ms. The fronto-central N2 typically peaks between 200 and 300 ms and is thought to reflect fast signaling of task relevant information and is modulated by conflict, task engagement and surprise (Ullsperger et al., 2014). The centro-parietal P3b is proposed to reflect evidence accumulation with regard to response selection (Twomey et al., 2015) and peaks around the time of the response in perceptual decision-making tasks (Ullsperger et al., 2014).

#### N2 amplitude

To test the effects of our experimental manipulation on N2 amplitude, we regressed perceptual certainty, deviant position and their interaction on N2. The model estimates are summarized in Table 3 and can be read out directly as mean differences in μV for main effects. Note that the N2 is a negative component; so negative estimates correspond to an increase in amplitude and positive estimates to a decrease in amplitude.

**Table 3:** Effects of perceptual certainty and deviant position on N2 amplitude.

**Variable**	***b***	***SE***	***t*-value**	***p*-value**	
Intercept	−3.42	0.32	−10.69	<0.001	^***^
PC i - b	−0.87	0.12	−7.57	<0.001	^***^
DP r - l	0.31	0.08	3.71	<0.001	^***^
PC: DP	0.24	0.09	2.60	0.009	^**^
**Variance components**	***SD***	**Goodness of fit**			
Participants	1.91	Log likelihood		−131736	
PC	0.48	REML deviance		263471	
DP	0.44				
Stimulus	0.41				
PC	0.30				
Residual	4.81				

*PC, Perceptual Certainty (intact – blurred); DP, Deviant Position (right – left)*.

*** *p < 0.001*,

** *p < 0.01*.

**Figure 5:**
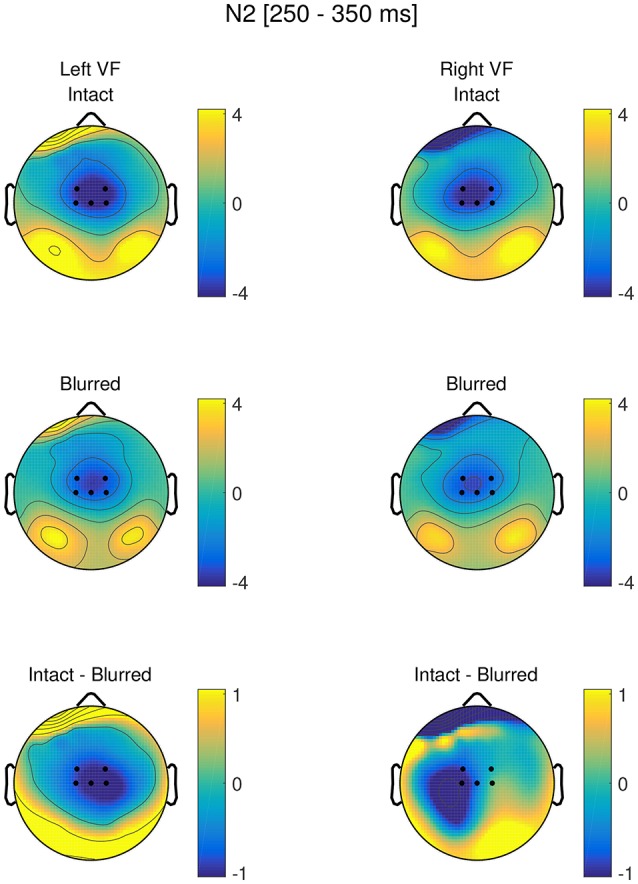
Topographies in the N2 time range (250–350 ms) for intact and blurred stimuli in the left and right visual field, as well as respective difference topographies. ROI electrodes are highlighted.

**Figure 6:**
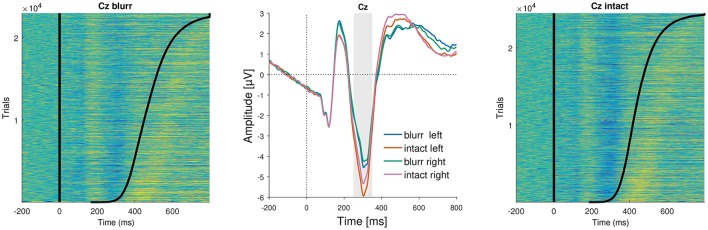
ERP images and average ERPs at electrode Cz. **(Left** and **Right)** Show color-coded amplitudes for all trials of blurr and intact, respectively. Trials are sorted according to RTs, marked as a black line. **(Center)** Average ERPs by perceptual certainty and visual field.

Figure 5 shows the topographies separately for blurred and intact stimuli in the left and right visual field, as well as the difference between blurred and intact stimuli in the left and right visual field, respectively. The time course at Cz is visualized in Figure 6.

We observed a significant main effect of perceptual certainty, with a reduced N2 for blurred compared to intact stimuli. Further, N2 amplitude was significantly reduced for the right compared to the left deviant position. Finally, we observed a significant interaction between perceptual certainty and deviant position.

To follow up this interaction, we computed models with the factors nested within each other. Those revealed a significant effect of perceptual certainty for the right (*b* = −0.77, *p* < 0.001), and the left deviant position (*b* = −1.01, *p* < 0.001). The descriptively larger effect estimate for the left target position, consistent with behavioral findings, suggests that the effect of perceptual certainty was stronger when the deviant was presented in the left visual field than when it was presented in the right visual field. Testing deviant position effects nested within blurred and intact stimuli, we observed a significant deviant position effect for intact (*b* = 0.43, *p* < 0.001), but only a trend for blurred stimuli (*b* = 0.19, *p* = 0.051). Note that these comparisons can alternatively be obtained using the difflsmeans function of the lmerTest package.

We ran ANOVA and regression for comparison. The results are comparable across methods (see F00 for detailed outputs). Comparing AICs and BICs, again suggests a better fit of the LMM compared to the regression (AIC: 263499 vs. 270239, BIC: 263621 vs. 270282).

#### P3b

**Figure 7:**
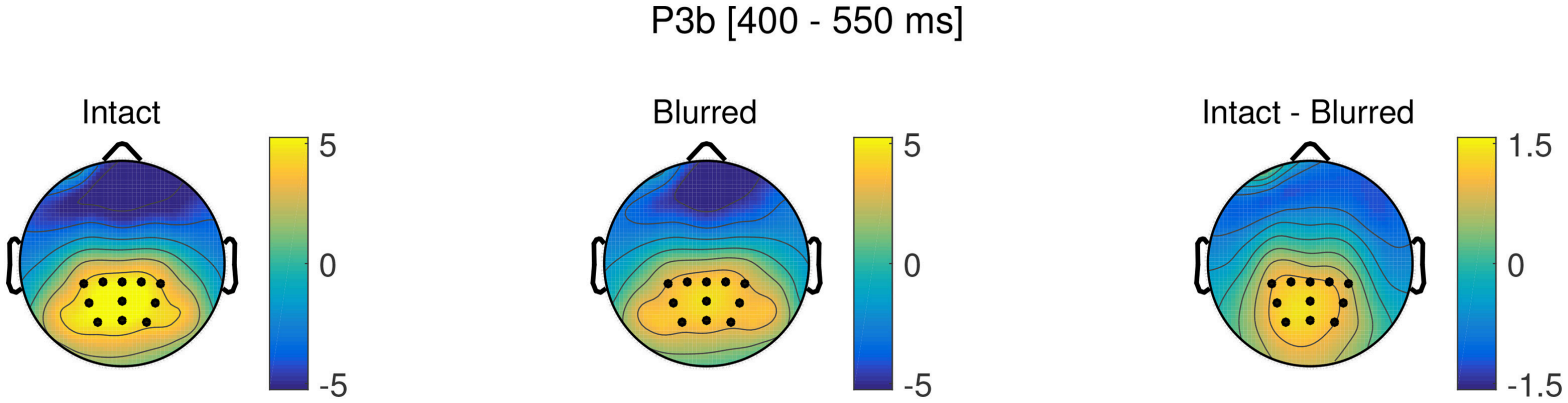
Topographies in the P3b time window (400–550 ms) for intact and blurred stimuli, as well as the difference topography. ROI electrodes are marked as dots.

**Figure 8:**
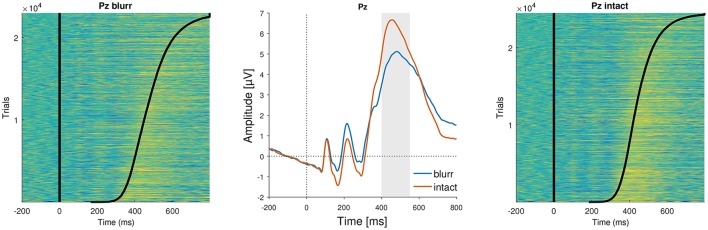
ERP images and average ERPs at electrode Pz. **(Left** and **Right)** Show color-coded amplitudes for all trials of blurr and intact, respectively. Trials are sorted according to RTs, marked as a black line. **(Center)** Average ERPs by perceptual certainty.

We next tested the effects of our experimental manipulations, perceptual certainty and deviant position, on P3b amplitude. The model revealed a significant main effect of perceptual certainty, that is P3b amplitude was reduced for blurred compared to intact stimuli. Topographies for blurred and intact stimuli, as well as the difference are displayed in Figure 7 and the time course is visualized in Figure 8. There was no significant main effect of or interaction with deviant position. We therefore reduced the model step-wise, first excluding the non-significant interaction and then excluding deviant position altogether. Model comparison favored the reduced model with perceptual certainty only, Δ$X_{\left(2\right)}^{2}$ = 0.79, *p* = 0.673 (ΔAIC = −3, ΔBIC = −20). However, note that we maintained random slopes for deviant position for participants, as removing this variance component significantly decreased model fit. This indicates that while there is no group level effect of deviant position, there are reliable individual differences in this effect, which might reflect differences in the use of top-down information for decision-making. The reduced model including fixed and random effects estimates is summarized in Table 4.

**Table 4:** Effects of perceptual certainty and deviant position on P3b amplitude.

**Variable**	***b***	***SE***	***t*-value**	***p*-value**	
Intercept	4.20	0.51	8.27	<0.001	^***^
PC i - b	1.17	0.17	6.67	<0.001	^***^
**Variance components**	***SD***	**Goodness of fit**			
Participants	3.17	Log likelihood		−128756	
PC	0.53	REML deviance		257513	
DP	0.46				
Stimulus	0.59				
PC	0.59				
Residual	4.49				

*PC, Perceptual Certainty (intact – blurred); DP, Deviant Position (right – left)*.

*** *p < 0.001*.

Comparing the results of ANOVA, regression and LMM, all methods converged to the same results. However, comparing fit indices, LMMs again suggested to better account for the data than ordinary regression (AIC: 257536 vs. 275627, BIC: 257641 vs. 275654).

#### Brain behavior relationship

So far, we established that showing blurred vs. intact objects in visual search affects performance, N2, and P3b. Furthermore, N2 and behavior are jointly affected by deviant position in interaction with perceptual certainty. Next, we tested whether behavior varies as a function of N2 and P3b amplitudes.

We tested the joint effects of N2 and P3b on accuracy and RTs, regressing their centered amplitudes, perceptual certainty, and deviant position on accuracy and RTs. For these analyses, we divided all single trial amplitudes by 10, as lme4 suggested rescaling of the variables to support model identifiability. Thus, the estimates from these analyses relate to amplitude changes of 10 μV. For accuracy, the full model including all predictors and their interactions revealed no significant 3-way interactions or 4-way interaction, also, there were no significant interactions of perceptual certainty with deviant position or N2. Exclusion of these interaction terms did not significantly decrease model fit, Δ$X_{\left(7\right)}^{2}$ = 2.60, *p* = 0.920, and fit indices were smaller for the reduced model (ΔAIC = −11, ΔBIC = −73). Model estimates are summarized in Table 5.

**Table 5:** Joint effects of N2 and P3b amplitude, perceptual certainty and deviant position on performance.

	**Accuracy**					**Reaction time**				
**Variable**	***b***	***SE***	***z*-value**	***p*-value**		***b***	***SE***	***t*-value**	***p*-value**	
Intercept	3.35	0.19	17.5	<0.001	^***^	470.00	9.23	50.92	<0.001	^***^
PC i - b	0.94	0.13	6.93	<0.001	^***^	−30.14	5.43	−5.55	<0.001	^***^
DP r - l	0.30	0.11	2.81	<0.001	^***^	−6.15	3.59	−1.71	0.093	
N2	−0.01	0.04	−0.31	0.758		13.04	2.26	5.77	<0.001	^***^
P3	0.69	0.05	13.82	<0.001	^***^	−32.23	0.94	−34.30	<0.001	^***^
PC:P3	0.25	0.09	2.69	0.007	^**^	6.78	1.78	3.81	<0.001	^***^
DP:P3	−0.21	0.09	−2.37	0.018	^*^	1.38	1.80	0.76	0.444	
N2:P3	0.18	0.07	2.46	0.014	^*^	−8.23	1.48	−5.54	<0.001	^***^
DP:N2	0.24	0.08	2.86	0.004	^**^	−7.37	1.74	−4.24	<0.001	^***^
DP:N2:P3	–	–	–	–		6.72	2.62	2.57	0.010	^*^
**Variance components**	***SD***	**Goodness of fit**				***SD***	**Goodness of fit**			
Participants	0.92	Log likelihood		−8706		49.92	Log likelihood		−256820	
PC	0.40	REML deviance		17413		14.62	REML deviance		513641	
DP	0.42				18.63					
N2	–				13.02					
Stimulus	0.47				19.04					
PC	0.40				19.33					
DP	0.24				7.46					
Residual					86.60					

*PC, Perceptual Certainty (intact – blurred); DP, Deviant Position (right – left)*.

*** *p < 0.001*,

** *p < 0.01*,

* *p < 0.05*.

There was no main effect of N2 on accuracy, but a significant interaction of N2 and deviant position. To follow up on this effect, we computed a nested model to obtain estimates of N2 effects separately for left and right deviant positions. While for the left deviant position, *larger* N2 amplitudes significantly related to higher detection likelihood (*b* = −0.13, *p* = 0.022), for the right deviant position, there was no significant association—if anything, *smaller* N2 tended to predict higher detection likelihood (*b* = 0.11, *p* = 0.086). Accuracy further increased with increasing P3b amplitude. Nested models to follow up the significant interactions of P3b with perceptual certainty and deviant position, respectively, showed significant P3b effects for intact (*b* = 0.82, *p* < 0.001) and blurred stimuli (*b* = 0. 57, *p* < 0.001), as well as deviants in the left (*b* = 0.80, *p* < 0.001) and right visual field (*b* = 0.59, *p* < 0.001). The overall effect of P3b amplitude on accuracy and the interaction with perceptual certainty are consistent with previous findings proposing a scaling of P3b amplitude with choice confidence (Boldt and Yeung, 2015) and the interpretation of P3b as reflecting evidence accumulation (Ullsperger et al., 2014; Murphy et al., 2015; Twomey et al., 2015). We further observed a significant interaction of N2 and P3b, that is, accuracy increased more strongly with P3b when N2 was smaller. The logistic regression we ran in comparison obtained similar results overall with the exception that it did not show a significant interaction of P3b and perceptual certainty. Again, fit indices were smaller for the GLMM compared to the logistic regression (AIC: 17454 vs. 19143, BIC: 17638 vs. 19222). We ran additional GLMMs, sequentially omitting random effects per item and random slopes, to see what produces the difference between the two methods. The estimate decreased when omitting the crossed random structure and was no longer significant in the model with random intercept per participants only. Thus this effect was revealed when controlling for variance in effects across participants.

**Figure 9:**
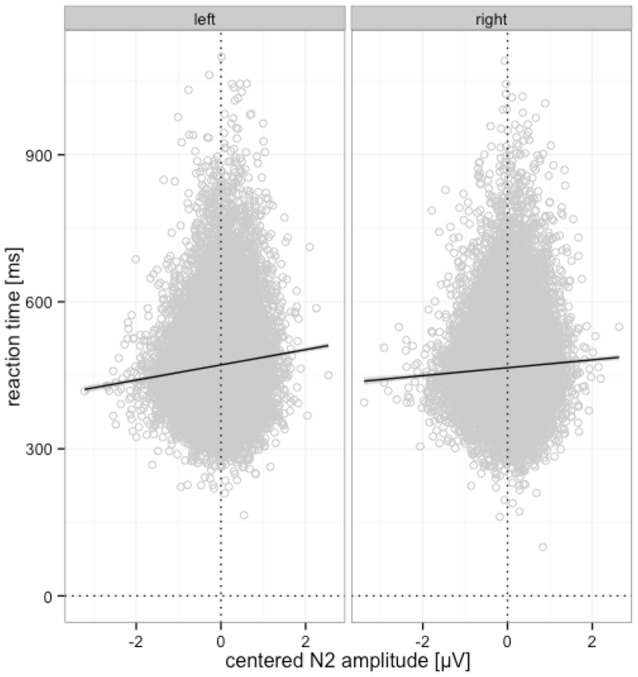
Relationship between N2 amplitude and reaction time for left and right deviant position. Predicted partial effects were computed with the remef package in R, the regression line is retrieved from a local linear model fit to the data points for illustration.

In the full model on RTs we observed no significant 2-way or higher order interactions between perceptual certainty and N2, so we excluded those, which did not significantly reduce model fit, Δ$X_{\left(6\right)}^{2}$ = 7.13, *p* = 0.309, and fit indices were smaller for the reduced model (ΔAIC = −5, ΔBIC = −57). RTs significantly decreased with increasing N2 amplitude. This effect was significant for deviants in the left (*b* = 16.73, *p* < 0.001), and the right visual field (*b* = 9.36, *p* < 0.001), as revealed with nested models to follow up the interaction of N2 with deviant position. The partial effects of this interaction on RTs, as retrieved using remef (Hohenstein and Kliegl, 2014), are displayed in Figure 9.

**Figure 10:**
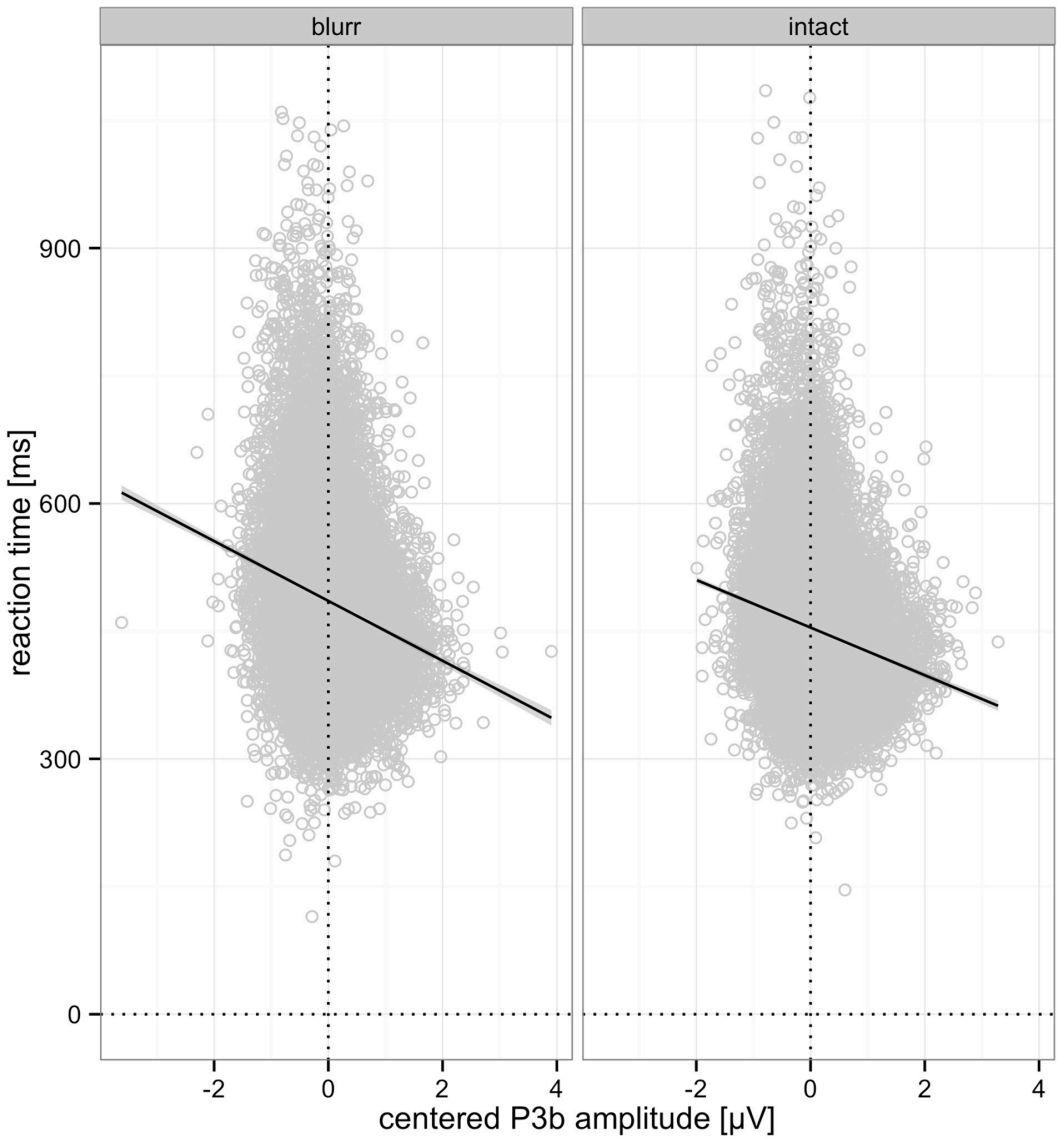
Relationship between centered P3b amplitude and RTs for blurred and intact stimuli. Predicted partial effects were computed with the remef package in R, the regression line is retrieved from a local linear model fit to the data points for illustration.

Moreover, we observed significantly shorter RTs for larger P3b amplitudes. This effect was significant for blurred (*b* = −35.62, *p* < 0.001) and intact stimuli (*b* = −28.84, *p* < 0.001), as obtained with a nested model to follow up the interaction. This effect is visualized in Figure 10.

**Figure 11:**
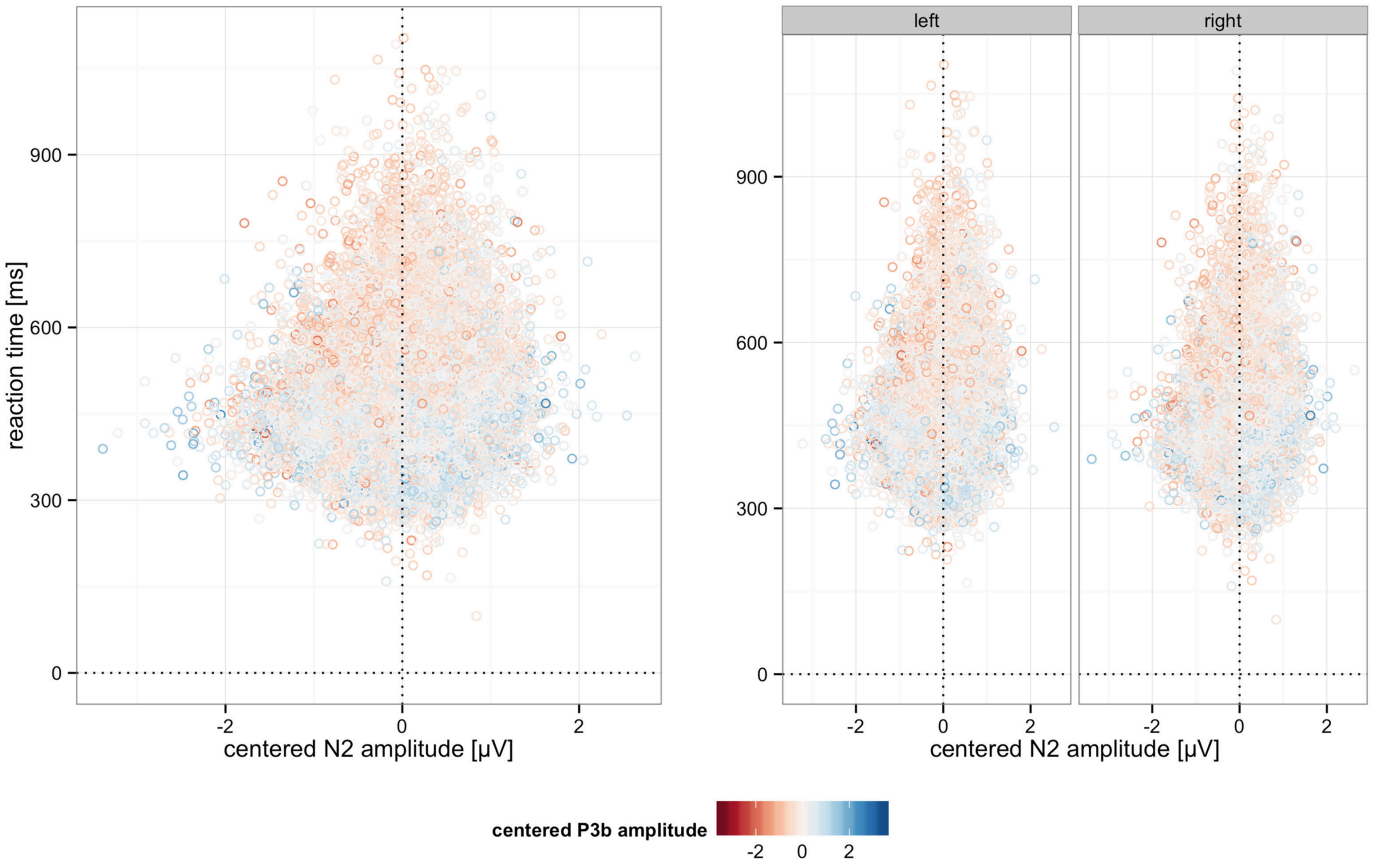
Interaction effects on RT. **(Left)** Interaction of P3b and N2 amplitude on RT. **(Right)** Interaction of P3b and N2 amplitude on RT by deviant position. In both panels, P3b amplitude is color-coded. Note that ERPs are centered and amplitudes are divided by ten. Random effects and the fixed effect of perceptual certainty were removed using remef.

In addition to these effects we observed significant interactions of N2 and P3b, as well as a significant 3-way interaction with deviant position. The N2 by P3b interaction suggests a stronger RT decrease with P3b amplitude increase when N2 amplitude is smaller (less negative). This interaction was significant for left (*b* = −11.59, *p* < 0.001) and right deviants (*b* = −4.86, *p* = 0.015), as revealed by a nested model. These effects, visualized in Figure 11, suggest complementary mechanisms underlying successful performance reflected in N2 and P3b. While both support faster performance, N2 amplitude seems to relate more tightly to the extraction of perceptual information, while P3b appears to relate more to the use or integration of given information for decision-making.

A regression ran as comparison obtained significant effects for all terms except for the 3-way interaction, in contrast to the LMM, the deviant position effect and the deviant position by P3b Interaction were significant. Moreover, fit indices favored LMM over regression (AIC: 513694 vs. 527163, BIC: 513929 vs. 527258). Again, to follow up the differences between regression and LMM results, we reduced the random structure of the LMM. When omitting crossed random effects, the deviant position effect became significant, as in the regression. Further omitting random slopes per participant rendered the 3-way interaction to a non-significant trend. However, even in the random intercept only model, we did not obtain a significant deviant position by P3b interaction, suggesting that this effect in the regression is produced by random intercept variance (note that even in the regression the estimate is only 3.49, which is very small even though it's twice the size of the LMM estimate).

To summarize, using single trial based LMM analyses, we obtained mostly comparable results to ANOVA and regression. When results differed, LMMs were more sensitive than ANOVAs and both more sensitive and more specific than regressions. The brain behavior analyses were further only applicable with single trial ERPs and hence LMMs and regression. Here, using continuous predictors, as for categorical predictors in the other analyses, LMMs outperformed ordinary regression.

## Discussion

The present study illustrates the advantages of single trial based analyses of EEG and behavioral data. As we could show, the ERP components meaningfully and differentially relate to trial-by-trial variations in behavior beyond variability caused by our experimental manipulations. This would not have been revealed using a traditional averaging approach. Therefore, while the present analyses are of exploratory nature, they highlight the flexibility of single-trial-based approaches in general and demonstrate the applicability of our processing pipeline in particular.

### (When) should you use this approach?

Why would you use complicated single-trial based LMM analyses of ERP data in simple orthogonal designs? As outlined in the introduction, ERP data often lack equal observations per cell, and individual differences in effect sizes, potentially biasing group estimates, are overlooked in averaging approaches. Further, as is well established in psycholinguistics, different stimuli can vary in characteristics unrelated to the experimental manipulations that might confound the effects of interest. As outlined by Baayen et al. (2008), this is not only true for words, but all naturalistic stimuli randomly drawn from a large population, such as objects, faces, artifacts or scenes. Thus, LMMs with crossed random effects would benefit every study using naturalistic stimuli.

While so far this pipeline has only been used for the analyses of distinct time windows (Frömer et al., 2016a,b; Fröber et al., 2017), the resulting data structure also allows for multiple robust regression on multiple time points to analyze the time course of effects (Hauk et al., 2006, 2009; Fischer and Ullsperger, 2013). However, bear in mind that only LMMs simultaneously account for random effects and might as well be conducted at multiple time points and electrodes. However then, robust estimates of Type I error need to be assessed. Statistical significance for LMMs can also be estimated using Markov chain Monte Carlo (MCMC) sampling, which would be more appropriate for multiple comparisons (Baayen et al., 2008).

Experimenters are encouraged to use parts of this pipeline according to their needs and personal taste. For instance, while we prefer procedures other than ICA for ocular correction and objective thresholds over investigator-dependent subjective data cleaning procedures (that might sometimes be more accurate and sometimes less), others might want to use a different preprocessing routine and only use some of the other parts of the pipeline. The modular way the pipeline is set up allows for flexibly swapping components for other approaches.

### Limitations

While the present approach circumvents some of the problems of traditional averaging approaches, it is still subject to others, such as component overlap. Specifically, and a problem of all methods applying statistical tests of multiple variables on local ERP distributions (e.g., mean amplitude at a ROI or peak amplitudes), the statistically observed effects are not necessarily distributed the way the ERP component of interest is. Statistically reliable effects might as well stem from a spatially overlapping different ERP component (C. B. Holroyd, 2015, personal communication). For LMM analyses, a simple proof of principle is to run the final model on all electrodes and to plot the topography of the fixed effects estimates to visually examine whether they show the expected distribution. More sophisticated approaches, in the time domain on a single electrode rather than in the spatial domain at a given time window, have been described by Smith and Kutas (2015b).

While the cluster based permutation approach is not subject to this limitation, its present implementation is only applicable to categorical variables with few factor levels. For screening and determining relevant time windows and recording sites, this problem could be circumvented by constructing median splits for parametric variables of interest and testing the main effects based on those categorical factors. However, as discussed in the introduction, this approach reduces statistical power and assumes linear scaling of the effects under investigation, which experimenters should bear in mind (Cohen, 1983; MacCallum et al., 2002; Baayen, 2004). Further, CBPT as implemented here operates on participant averages and therefore holds the same problems as other approaches aggregating within subjects and conditions first. Therefore, results from CBPT might differ from those obtained using LMMs with a better control of additional sources of variance. Last but not least, the CBPT is rather conservative in some cases, such as small, local effects (Luck and Gaspelin, 2017). However, it can be a valuable tool to objectively narrow down the amount of data to submit to further analyses and thereby decrease *investigator degrees of freedom* and the risk of Type I errors. An extension of this approach to single-trial based regression (possibly LMM) analyses would be a valuable methodological contribution to robust effect estimation and future research.

## Conclusion

The present processing pipeline integrates open source toolboxes for EEG data processing, EEGLAB (Delorme and Makeig, 2004) and FieldTrip (Oostenveld et al., 2011), and statistical analyses, lme4 (Bates et al., 2015b). It uses a single-trial regression based approach, circumventing limitations of traditional averaging approaches, while trying to maintain objectivity with regard to what data the analyses are applied to and thereby reducing investigator degrees of freedom. While some limitations remain, we consider this approach a major improvement compared to traditional ERP approaches and a good starting point for the development of even better analysis tools.

## Author contributions

RF collected data. RF and MM wrote analysis scripts. RF, MM, and RA designed the experiment, discussed data, and contributed to the manuscript.

## Ethics statement

This study was carried out in accordance with the recommendations of the ethics committee of the Psychology department of Humboldt-Universität zu Berlin with written informed consent from all subjects. All subjects gave written informed consent in accordance with the Declaration of Helsinki. The protocol was approved by the ethics committee of the Psychology department of Humboldt-Universität zu Berlin.

### Conflict of interest statement

The authors declare that the research was conducted in the absence of any commercial or financial relationships that could be construed as a potential conflict of interest.

## References

[B1] Amsel B. D. (2011). Tracking real-time neural activation of conceptual knowledge using single-trial event-related potentials. Neuropsychologia 49, 970–983. 10.1016/j.neuropsychologia.2011.01.003 21219919

[B2] Baayen R. H. (2004). Statistics in psycholinguistics: a critique of some current gold standards, in Mental Lexicon Working Papers, eds Libben G. Nault K. (Edmonton, CA: Mental Lexicon Research Project), 1–47.

[B3] Baayen R. H. Davidson D. J. Bates D. M. (2008). Mixed-effects modeling with crossed random effects for subjects and items. J. Mem. Lang. 59, 390–412. 10.1016/j.jml.2007.12.005

[B4] Bagiella E. Sloan R. P. Heitjan D. F. (2000). Mixed-effects models in psychophysiology. Psychophysiology 37, 13–20. 10.1111/1469-8986.3710013 10705763

[B5] Barr D. J. Levy R. Scheepers C. Tily H. J. (2013). Random effects structure for confirmatory hypothesis testing: keep it maximal. J. Mem. Lang. 68, 255–278. 10.1016/j.jml.2012.11.001 24403724 PMC3881361

[B6] Bates D. Kliegl R. Vasishth S. Baayen H. (2015a). Parsimonious mixed models. arXiv:1506.04967v1

[B7] Bates D. Mächler M. Bolker B. Walker S. (2015b). Fitting linear mixed-effects models using lme4. J. Stat. Soft 67, 1–48. 10.18637/jss.v067.i01

[B8] Boldt A. Yeung N. (2015). Shared neural markers of decision confidence and error detection. J. Neurosci. 35, 3478–3484. 10.1523/JNEUROSCI.0797-14.2015 25716847 PMC4339357

[B9] Bolker B. M. (ed.). (2008). Standard statistics revisited, in Ecological Models and Data in R (Princeton, NJ: Princeton University Press), 298–312.

[B10] Cavanagh J. F. Figueroa C. M. Cohen M. X. Frank M. J. (2012). Frontal theta reflects uncertainty and unexpectedness during exploration and exploitation. Cereb. Cortex 22, 2575–2586. 10.1093/cercor/bhr332 22120491 PMC4296208

[B11] Cohen J. (1983). The cost of dichotomization. Appl. Psychol. Meas. 7, 249–253. 10.1177/014662168300700301

[B12] Collins A. G. E. Frank M. J. (2016). Neural signature of hierarchically structured expectations predicts clustering and transfer of rule sets in reinforcement learning. Cognition 152, 160–169. 10.1016/j.cognition.2016.04.002 27082659 PMC5595218

[B13] Cronbach L. J. (1957). The two disciplines of scientific psychology. Amer. Psychol. 12, 671–684. 10.1037/h0043943

[B14] Dambacher M. Kliegl R. Hofmann M. Jacobs A. M. (2006). Frequency and predictability effects on event-related potentials during reading. Brain Res. 1084, 89–103. 10.1016/j.brainres.2006.02.010 16545344

[B15] Delorme A. Makeig S. (2004). EEGLAB: an open source toolbox for analysis of single-trial EEG dynamics including independent component analysis. J. Neurosci. Methods 134, 9–21. 10.1016/j.jneumeth.2003.10.009 15102499

[B16] Dimigen O. Sommer W. Hohlfeld A. Jacobs A. M. Kliegl R. (2011). Coregistration of eye movements and EEG in natural reading: analyses and review. J. Exp. Psychol. Gen. 140, 552–572. 10.1037/a0023885 21744985

[B17] Fischer A. G. Ullsperger M. (2013). Real and fictive outcomes are processed differently but converge on a common adaptive mechanism. Neuron 79, 1243–1255. 10.1016/j.neuron.2013.07.006 24050408

[B18] Fröber K. Stürmer B. Frömer R. Dreisbach G. (2017). The role of affective evaluation in conflict adaptation: an LRP study. Brain Cogn. 116, 9–16. 10.1016/j.bandc.2017.05.003 28570905

[B19] Frömer R. Stürmer B. Sommer W. (2016a). The better, the bigger: the effect of graded positive performance feedback on the reward positivity. Biol. Psychol. 114, 61–68. 10.1016/j.biopsycho.2015.12.011 26756995

[B20] Frömer R. Stürmer B. Sommer W. (2016b). (Don't) Mind the effort: effects of contextual interference on ERP indicators of motor preparation. Psychophysiology 53, 1577–1586. 10.1111/psyp.12703 27383866

[B21] Hauk O. Davis M. H. Ford M. Pulvermüller F. Marslen-Wilson W. D. (2006). The time course of visual word recognition as revealed by linear regression analysis of ERP data. Neuroimage 30, 1383–1400. 10.1016/j.neuroimage.2005.11.048 16460964

[B22] Hauk O. Pulvermüller F. Ford M. Marslen-Wilson W. D. Davis M. H. (2009). Can I have a quick word*?* Early electrophysiological manifestations of psycholinguistic processes revealed by event-related regression analysis of the EEG. Biol. Psychol. 80, 64–74. 10.1016/j.biopsycho.2008.04.015 18565639

[B23] Hohenstein S. Kliegl R. (2014). remef (REMove Effects). version v0. 6.10.

[B24] Ille N. Berg P. Scherg M. (2002). Artifact correction of the ongoing EEG using spatial filters based on artifact and brain signal topographies. J. Clin. Neurophysiol. 19, 113–124. 10.1097/00004691-200203000-00002 11997722

[B25] Kliegl R. Hohenstein S. Yan M. McDonald S. A. (2013). How preview space/time translates into preview cost/benefit for fixation durations during reading. Q. J. Exp. Psychol. 66, 581–600. 10.1080/17470218.2012.658073 22515948

[B26] Kliegl R. Wei P. Dambacher M. Yan M. Zhou X. (2010). Experimental effects and individual differences in linear mixed models: estimating the relationship between spatial, object, and attraction effects in visual attention. Front. Psychol. 1:238. 10.3389/fpsyg.2010.00238 21833292 PMC3153842

[B27] Kornrumpf B. Dimigen O. Sommer W. (2017). Lateralization of posterior alpha EEG reflects the distribution of spatial attention during saccadic reading. Psychophysiology, 54, 809–823. 10.1111/psyp.12849 28240816

[B28] Kornrumpf B. Niefind F. Sommer W. Dimigen O. (2016). Neural correlates of word recognition: a systematic comparison of natural reading and rapid serial visual presentation. J. Cogn. Neurosci. 28, 1374–1391. 10.1162/jocn_a_00977 27167402

[B29] Luck S. J. Gaspelin N. (2017). How to get statistically significant effects in any ERP experiment (and why you shouldn't). Psychophysiology 54, 146–157. 10.1111/psyp.12639 28000253 PMC5178877

[B30] MacCallum R. C. Zhang S. Preacher K. J. Rucker D. D. (2002). On the practice of dichotomization of quantitative variables. Psychol. Methods 7, 19–40. 10.1037/1082-989X.7.1.19 11928888

[B31] Maier M. Glage P. Hohlfeld A. Abdel Rahman R. (2014). Does the semantic content of verbal categories influence categorical perception? An ERP study. Brain Cogn. 91, 1–10. 10.1016/j.bandc.2014.07.008 25163810

[B32] Maris E. (2004). Randomization tests for ERP topographies and whole spatiotemporal data matrices. Psychophysiology 41, 142–151. 10.1111/j.1469-8986.2003.00139.x 14693009

[B33] Maris E. (2012). Statistical testing in electrophysiological studies. Psychophysiology 49, 549–565. 10.1111/j.1469-8986.2011.01320.x 22176204

[B34] Maris E. Oostenveld R. (2007). Nonparametric statistical testing of EEG- and MEG-data. J. Neurosci. Methods 164, 177–190. 10.1016/j.jneumeth.2007.03.024 17517438

[B35] Matuschek H. Kliegl R. Vasishth S. Baayen H. Bates D. (2017). Balancing Type I error and power in linear mixed models. J. Mem. Lang. 94, 305–315. 10.1016/j.jml.2017.01.001

[B36] Murphy P. R. Robertson I. H. Harty S. O'Connell R. G. (2015). Neural evidence accumulation persists after choice to inform metacognitive judgments. Elife 4:e11946. 10.7554/eLife.11946 26687008 PMC4749550

[B37] Oostenveld R. Fries P. Maris E. Schoffelen J. M. (2011). FieldTrip: open source software for advanced analysis of MEG, EEG, and invasive electrophysiological data. Comput. Intell. Neurosci. 2011:156869. 10.1155/2011/156869 21253357 PMC3021840

[B38] Pinheiro J. Bates D. M. (2000). Mixed Effects Models in S and S-PLUS. New York, NY: Springer 10.1007/978-1-4419-0318-1

[B39] R Core Team (2014). R: A Language and Environment for Statistical Computing. Vienna: R Foundation for Statistical Computing Available online at: http://www.R-project.org/

[B40] Smith N. J. Kutas M. (2015a). Regression-based estimation of ERP waveforms: I. The rERP framework. Psychophysiology 52, 157–168. 10.1111/psyp.12317 25141770 PMC5308234

[B41] Smith N. J. Kutas M. (2015b). Regression-based estimation of ERP waveforms: II. Nonlinear effects, overlap correction, and practical considerations. Psychophysiology 52, 169–181. 10.1111/psyp.12320 25195691 PMC5306445

[B42] Twomey D. M. Murphy P. R. Kelly S. P. O'Connell R. G. (2015). The classic P300 encodes a build-to-threshold decision variable. Eur. J. Neurosci. 42, 1636–1643. 10.1111/ejn.12936 25925534

[B43] Ullsperger M. Fischer A. G. Nigbur R. Endrass T. (2014). Neural mechanisms and temporal dynamics of performance monitoring. Trends Cogn. Sci. 18, 259–267. 10.1016/j.tics.2014.02.009 24656460

